# Supplementary material for: Intracellular Diversity of WNV within Circulating Avian Peripheral Blood Mononuclear Cells Reveals Host-Dependent Patterns of Polyinfection
Source: Pathogens. 2023 May 26;12(6):767. doi: 10.3390/pathogens12060767 (PMC10300861; doi:10.3390/pathogens12060767)
Supplement: Supplementary file 1 [file pathogens-12-00767-s001.zip › pathogens-2341594-supplementary.pdf]

Supplementary Methods

Establishing an Amplicon Targeted Sequencing Approach

To overcome sequencing errors and PCR-mediated recombination that might introduce biases (1-4), we adjusted a library preparation strategy established by others (5). This strategy offers the inclusion of a stretch of degenerate nucleotides included into the cDNA synthesis primer that enables "tagging" of the input cDNA template before proceeding on to PCR amplification steps. This stretch of nucleotides served as an identifier sequence and was previously referred to as "Primer ID"(5).

Since the focus of this study was to identify intra-host viral genetic diversity and we specifically aimed at sequencing single cells carrying a plethora of viral genomes, it was important to be able to identify low frequency genomes and to have as low sequencing bias as possible The inclusion of the Primer ID sequence allowed each original template copy to be tagged with an identifier sequence. Finding multiple identical Primer ID sequences during the analysis process of the sequencing products indicated that genomes tagged with the same Primer ID originated from a single original input template. These were subsequently collapsed to create a consensus sequence for each original template.

We adapted the Primer ID approach to the Illumina MiSeq platform and amplified a 335 bp long fragment within the WNV genome that included the barcoded region within the NS4b segment (Supplementary Table, table of Illumina barcodes used for this study, excel sheet table-Illumina barcodes used for sample multiplexing (6). Library preparation is further detailed in the Experimental procedures section. Using the Primer ID method we were able to generate libraries from an input of as little as 50 viral genomes.

Supplementary Table S1. Table of Illumina barcodes.

Name	Index	Sequence
i5001	ctctctatacgt	AATGATACGGCGACCACCGAGATCTACACctctctatacgtTCGTCGGCAGCGTC
i5002	tatcctctacgt	AATGATACGGCGACCACCGAGATCTACACtatcctctacgtTCGTCGGCAGCGTC
i5003	gtaaggagacgt	AATGATACGGCGACCACCGAGATCTACACgtaaggagacgtTCGTCGGCAGCGTC
i5004	actgcataacgt	AATGATACGGCGACCACCGAGATCTACACactgcataacgtTCGTCGGCAGCGTC
i5005	aaggagtaacgt	AATGATACGGCGACCACCGAGATCTACACaaggagtaacgtTCGTCGGCAGCGTC
i5006	ctaagcctacgt	AATGATACGGCGACCACCGAGATCTACACctaagcctacgtTCGTCGGCAGCGTC
i5007	cgtctaatacgt	AATGATACGGCGACCACCGAGATCTACACcgtctaatacgtTCGTCGGCAGCGTC
i5008	tctctccgacgt	AATGATACGGCGACCACCGAGATCTACACtctctccgacgtTCGTCGGCAGCGTC

i5009	tcgactagacgt	AATGATACGGCGACCACCGAGATCTACACtcgactagacgtTCGTCGGCAGCGTC
i5010	ttctagctacgt	AATGATACGGCGACCACCGAGATCTACACttctagctacgtTCGTCGGCAGCGTC
i5011	cctagagtacgt	AATGATACGGCGACCACCGAGATCTACACcctagagtacgtTCGTCGGCAGCGTC
i5012	gcgtaagaacgt	AATGATACGGCGACCACCGAGATCTACACgcgtaagaacgtTCGTCGGCAGCGTC
i5013	ctattaagacgt	AATGATACGGCGACCACCGAGATCTACACctattaagacgtTCGTCGGCAGCGTC
i5014	aaggctatacgt	AATGATACGGCGACCACCGAGATCTACACaaggctatacgtTCGTCGGCAGCGTC
i5015	gagccttaacgt	AATGATACGGCGACCACCGAGATCTACACgagccttaacgtTCGTCGGCAGCGTC
i5016	ttatgcgaacgt	AATGATACGGCGACCACCGAGATCTACACttatgcgaacgtTCGTCGGCAGCGTC
i5017	ctctctattgca	AATGATACGGCGACCACCGAGATCTACACctctctattgcaTCGTCGGCAGCGTC
i5018	tatcctcttgca	AATGATACGGCGACCACCGAGATCTACACtatcctcttgcaTCGTCGGCAGCGTC
i5019	gtaaggagtgca	AATGATACGGCGACCACCGAGATCTACACgtaaggagtgcaTCGTCGGCAGCGTC
i5020	actgcatatgca	AATGATACGGCGACCACCGAGATCTACACactgcatatgcaTCGTCGGCAGCGTC
i5021	aaggagtatgca	AATGATACGGCGACCACCGAGATCTACACaaggagtatgcaTCGTCGGCAGCGTC
i5022	ctaagccttgca	AATGATACGGCGACCACCGAGATCTACACctaagccttgcaTCGTCGGCAGCGTC
i5023	cgtctaattgca	AATGATACGGCGACCACCGAGATCTACACcgtctaattgcaTCGTCGGCAGCGTC
i5024	tctctccgtgca	AATGATACGGCGACCACCGAGATCTACACtctctccgtgcaTCGTCGGCAGCGTC
i5025	tcgactagtgca	AATGATACGGCGACCACCGAGATCTACACtcgactagtgcaTCGTCGGCAGCGTC
i5026	ttctagcttgca	AATGATACGGCGACCACCGAGATCTACACttctagcttgcaTCGTCGGCAGCGTC
i5027	cctagagttgca	AATGATACGGCGACCACCGAGATCTACACcctagagttgcaTCGTCGGCAGCGTC
i5028	gcgtaagatgca	AATGATACGGCGACCACCGAGATCTACACgcgtaagatgcaTCGTCGGCAGCGTC
i5029	ctattaagtgca	AATGATACGGCGACCACCGAGATCTACACctattaagtgcaTCGTCGGCAGCGTC
i5030	aaggctattgca	AATGATACGGCGACCACCGAGATCTACACaaggctattgcaTCGTCGGCAGCGTC
i5031	gagccttatgca	AATGATACGGCGACCACCGAGATCTACACgagccttatgcaTCGTCGGCAGCGTC
i5032	ttatgcgatgca	AATGATACGGCGACCACCGAGATCTACACttatgcgatgcaTCGTCGGCAGCGTC
i5033	ctctctatctag	AATGATACGGCGACCACCGAGATCTACACctctctatctagTCGTCGGCAGCGTC
i5034	tatcctctctag	AATGATACGGCGACCACCGAGATCTACACtatcctctctagTCGTCGGCAGCGTC
i5035	gtaaggagctag	AATGATACGGCGACCACCGAGATCTACACgtaaggagctagTCGTCGGCAGCGTC
i5036	actgcatactag	AATGATACGGCGACCACCGAGATCTACACactgcatactagTCGTCGGCAGCGTC
i5037	aaggagtactag	AATGATACGGCGACCACCGAGATCTACACaaggagtactagTCGTCGGCAGCGTC
i5038	ctaagcctctag	AATGATACGGCGACCACCGAGATCTACACctaagcctctagTCGTCGGCAGCGTC
i5039	cgtctaatctag	AATGATACGGCGACCACCGAGATCTACACcgtctaatctagTCGTCGGCAGCGTC
i5040	tctctccgctag	AATGATACGGCGACCACCGAGATCTACACtctctccgctagTCGTCGGCAGCGTC
i5041	tcgactagctag	AATGATACGGCGACCACCGAGATCTACACtcgactagctagTCGTCGGCAGCGTC
i5042	ttctagctctag	AATGATACGGCGACCACCGAGATCTACACttctagctctagTCGTCGGCAGCGTC
i5043	cctagagtctag	AATGATACGGCGACCACCGAGATCTACACcctagagtctagTCGTCGGCAGCGTC
i5044	gcgtaagactag	AATGATACGGCGACCACCGAGATCTACACgcgtaagactagTCGTCGGCAGCGTC
i5045	ctattaagctag	AATGATACGGCGACCACCGAGATCTACACctattaagctagTCGTCGGCAGCGTC
i5046	aaggctatctag	AATGATACGGCGACCACCGAGATCTACACaaggctatctagTCGTCGGCAGCGTC
i5047	gagccttactag	AATGATACGGCGACCACCGAGATCTACACgagccttactagTCGTCGGCAGCGTC
i5048	ttatgcgactag	AATGATACGGCGACCACCGAGATCTACACttatgcgactagTCGTCGGCAGCGTC
i5049	ctctctatgatc	AATGATACGGCGACCACCGAGATCTACACctctctatgatcTCGTCGGCAGCGTC
i5050	tatcctctgatc	AATGATACGGCGACCACCGAGATCTACACtatcctctgatcTCGTCGGCAGCGTC
i5051	gtaaggaggatc	AATGATACGGCGACCACCGAGATCTACACgtaaggaggatcTCGTCGGCAGCGTC
i5052	actgcatagatc	AATGATACGGCGACCACCGAGATCTACACactgcatagatcTCGTCGGCAGCGTC
i5053	aaggagtagatc	AATGATACGGCGACCACCGAGATCTACACaaggagtagatcTCGTCGGCAGCGTC
i5054	ctaagcctgatc	AATGATACGGCGACCACCGAGATCTACACctaagcctgatcTCGTCGGCAGCGTC
i5055	cgtctaatgatc	AATGATACGGCGACCACCGAGATCTACACcgtctaatgatcTCGTCGGCAGCGTC
i5056	tctctccggatc	AATGATACGGCGACCACCGAGATCTACACtctctccggatcTCGTCGGCAGCGTC
i5057	tcgactaggatc	AATGATACGGCGACCACCGAGATCTACACtcgactaggatcTCGTCGGCAGCGTC
i5058	ttctagctgatc	AATGATACGGCGACCACCGAGATCTACACttctagctgatcTCGTCGGCAGCGTC

i5059	cctagagtgatc	AATGATACGGCGACCACCGAGATCTACACcctagagtgatcTCGTCGGCAGCGTC
i5060	gcgtaagagatc	AATGATACGGCGACCACCGAGATCTACACgcgtaagagatcTCGTCGGCAGCGTC
i5061	ctattaaggatc	AATGATACGGCGACCACCGAGATCTACACctattaaggatcTCGTCGGCAGCGTC
i5062	aaggctatgatc	AATGATACGGCGACCACCGAGATCTACACaaggctatgatcTCGTCGGCAGCGTC
i5063	gagccttagatc	AATGATACGGCGACCACCGAGATCTACACgagccttagatcTCGTCGGCAGCGTC
i5064	ttatgcgagatc	AATGATACGGCGACCACCGAGATCTACACttatgcgagatcTCGTCGGCAGCGTC
i701	taaggcga	CAAGCAGAAGACGGCATACGAGATtaaggcgaGTCTCGTGGGCTCGG
i702	cgtactag	CAAGCAGAAGACGGCATACGAGATcgtactagGTCTCGTGGGCTCGG
i703	aggcagaa	CAAGCAGAAGACGGCATACGAGATaggcagaaGTCTCGTGGGCTCGG
i704	tcctgagc	CAAGCAGAAGACGGCATACGAGATtcctgagcGTCTCGTGGGCTCGG
i705	ggactcct	CAAGCAGAAGACGGCATACGAGATggactcctGTCTCGTGGGCTCGG
i706	taggcatg	CAAGCAGAAGACGGCATACGAGATtaggcatgGTCTCGTGGGCTCGG
i707	ctctctac	CAAGCAGAAGACGGCATACGAGATctctctacGTCTCGTGGGCTCGG
i708	cagagagg	CAAGCAGAAGACGGCATACGAGATcagagaggGTCTCGTGGGCTCGG
i709	gctacgct	CAAGCAGAAGACGGCATACGAGATgctacgctGTCTCGTGGGCTCGG
i710	cgaggctg	CAAGCAGAAGACGGCATACGAGATcgaggctgGTCTCGTGGGCTCGG
i711	aagaggca	CAAGCAGAAGACGGCATACGAGATaagaggcaGTCTCGTGGGCTCGG
i712	gtagagga	CAAGCAGAAGACGGCATACGAGATgtagaggaGTCTCGTGGGCTCGG

Supplementary Table S2. Table of primers for Illumina sample multiplexing and primer ID.

Name	Sequence
ID_cDNAWNV_7374_Rev	GTCTCGTGGGCTCGGAGATGTGTATAAGAGACAGNNNNNNNNNCAGTGCCATCCACTACAGCGTTCT
R1_5'_WNV_for	TCGTCGGCAGCGTCAGATGTGTATAAGAGACAGNNNNTTCCCCTTCGTCGATGTTGG
5'_ID_Primer_Rev	GTCTCGTGGGCTCGGAGATGTGTAT
Illumina index i5 (N5XX)	AATGATACGGCGACCACCGAGATCTACAC[i5]TCGTCGGCAGCGTC
Illumina index i7 (N7XXX)	CAAGCAGAAGACGGCATACGAGAT[i7]GTCTCGTGGGCTCGG

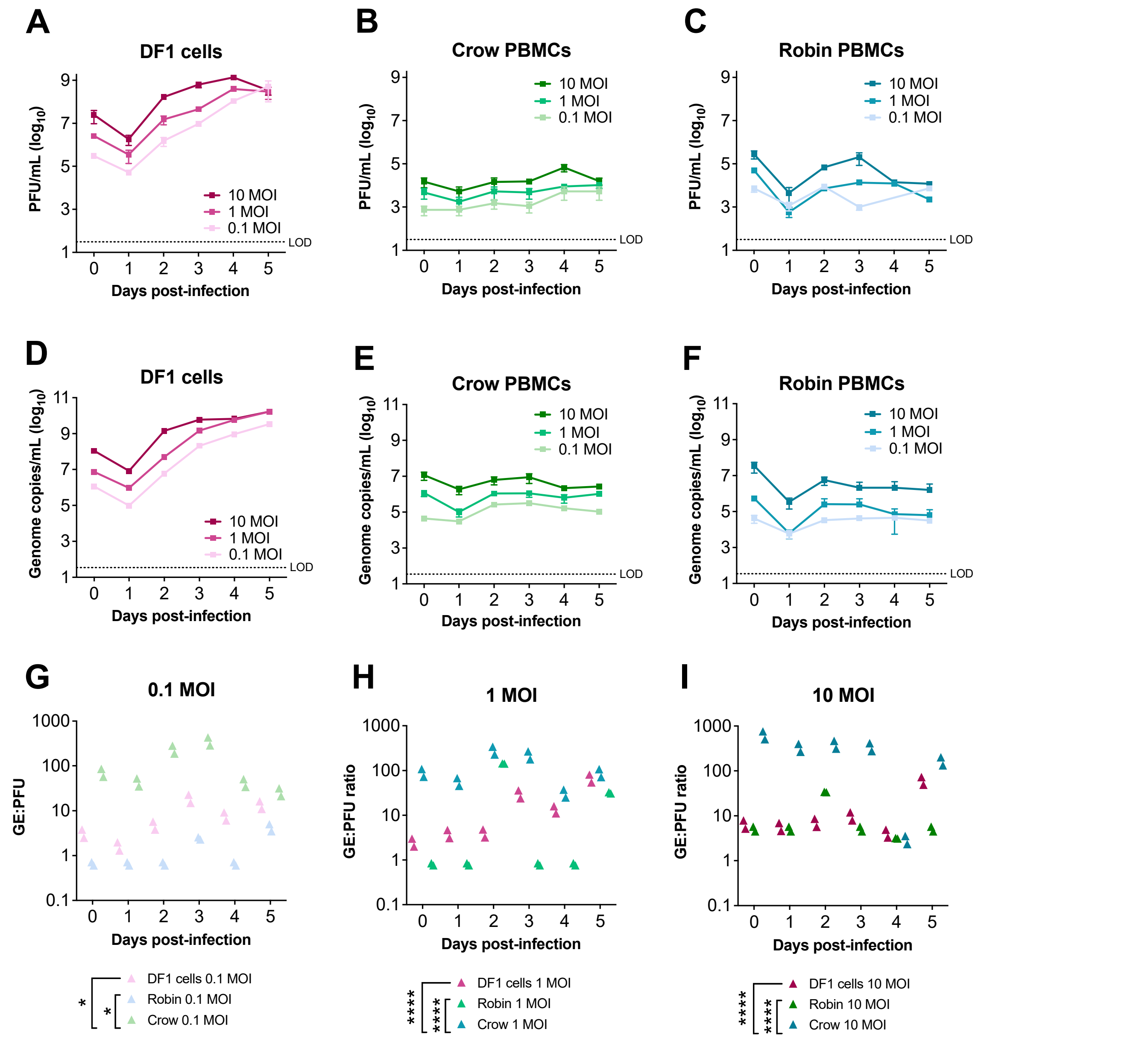

Supplementary Figure S1. WNV replicates in DF1 cells and *ex vivo* crow and robin PBMCs. Titers determined by plaque assay of DF1 cells (A), crow PBMCs (B) and robin PBMCs (C) infected with field strain WNV at MOI of 0.1, 1 and 10 (n=2 wells per cell type per MOI). Genome copies determined by qRT-PCR of DF1 cells (D), crow PBMCs (E) and robin PBMCs (F) (n=2 wells per cell type per MOI). WNV genome equivalents to plaque forming units (GE:PFU) at MOI of 0.1 (G) (*, DF1 vs crow PBMC, $P = 0.0215$; *, robin vs crow PBMC, $P = 0.0185$; 2-way ANOVA, Tukey's Multiple Comparison), 1 (H) (****, DF1 vs crow PBMC, $P < 0.0001$; ****, robin vs crow PBMC, $P < 0.0001$; 2-way ANOVA, Tukey's Multiple Comparison) and 10 (I) (****, DF1 vs crow PBMC, $P < 0.0001$; ****, robin vs crow PBMC, $P < 0.0001$; 2-way ANOVA, Tukey's Multiple Comparison) of DF1 cells, robin PBMCs and crow PBMCs.

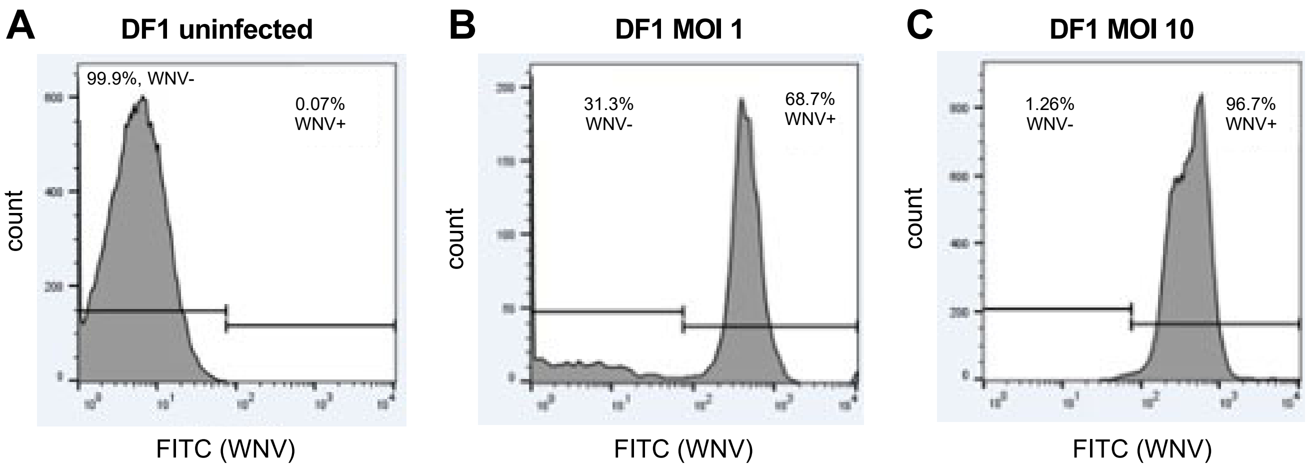

Supplementary Figure S2. A) uninfected, B) MOI 1 and C) MOI 10 WNV-infected DF1 cells were stained using a FITC labeled-anti-West Nile virus monoclonal antibody. Cells were analyzed by flow cytometry and gated as positive or negative based on FITC (WNV) signal.

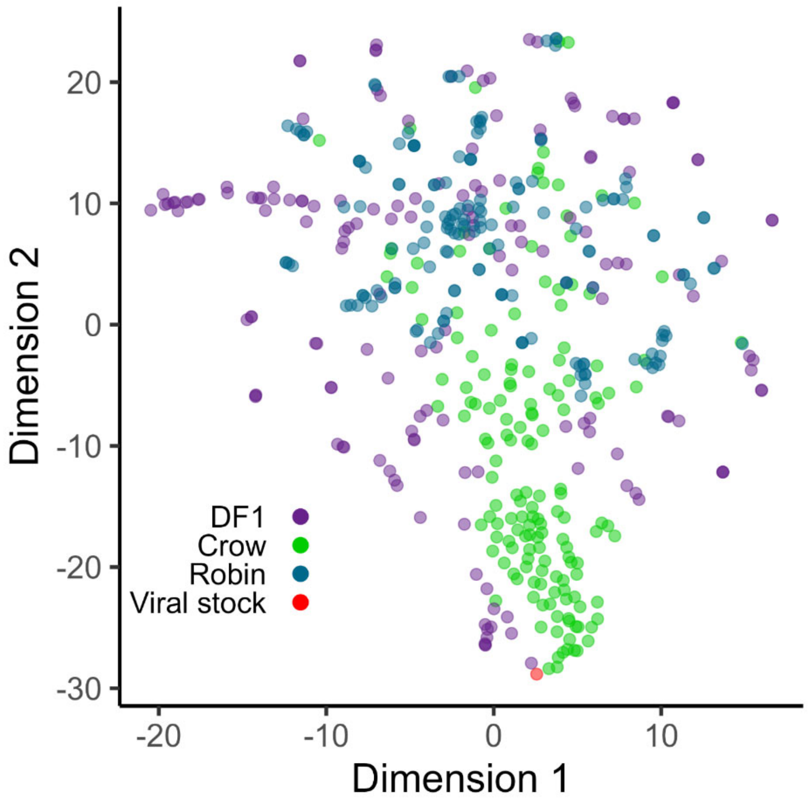

Supplementary Figure S3. T-Distributed Stochastic Neighbor Embedding (t-SNE) analysis to plot WNV barcode composition within individual cells in two-dimensional space.

References Cited

1. Gorzer I, Guelly C, Trajanoski S, Puchhammer-Stockl E. 2010. The impact of PCR-generated recombination on diversity estimation of mixed viral populations by deep sequencing. J Virol Methods 169:248-52.

2. Jabara CB, Jones CD, Roach J, Anderson JA, Swanstrom R. 2011. Accurate sampling and deep sequencing of the HIV-1 protease gene using a Primer ID. Proc Natl Acad Sci U S A 108:20166-71.
3. Keys JR, Zhou S, Anderson JA, Eron JJ, Jr., Rackoff LA, Jabara C, Swanstrom R. 2015. Primer ID Informs Next-Generation Sequencing Platforms and Reveals Preexisting Drug Resistance Mutations in the HIV-1 Reverse Transcriptase Coding Domain. AIDS Res Hum Retroviruses 31:658-68.
4. Meyerhans A, Vartanian JP, Wain-Hobson S. 1990. DNA recombination during PCR. Nucleic Acids Res 18:1687-91.
5. Zhou S, Jones C, Mieczkowski P, Swanstrom R. 2015. Primer ID Validates Template Sampling Depth and Greatly Reduces the Error Rate of Next-Generation Sequencing of HIV-1 Genomic RNA Populations. J Virol 89:8540-55.
6. Zanini F, Robinson ML, Croote D, Sahoo MK, Sanz AM, Ortiz-Lasso E, Albornoz LL, Rosso F, Montoya JG, Goo L, Pinsky BA, Quake SR, Einav S. 2018. Virus-inclusive single-cell RNA sequencing reveals the molecular signature of progression to severe dengue. Proc Natl Acad Sci U S A 115:E12363-E12369.

Disclaimer/Publisher's Note: The statements, opinions and data contained in all publications are solely those of the individual author(s) and contributor(s) and not of MDPI and/or the editor(s). MDPI and/or the editor(s) disclaim responsibility for any injury to people or property resulting from any ideas, methods, instructions or products referred to in the content.
